# Supplementary material for: Response Variability to Drug Testing in Two Models of Chemically Induced Colitis
Source: Int J Mol Sci. 2023 Mar 29;24(7):6424. doi: 10.3390/ijms24076424 (PMC10094987; doi:10.3390/ijms24076424)
Supplement: Supplementary file 1 [file ijms-24-06424-s001.zip › ijms-2296622-supplementary.pdf]

Supplementary figures

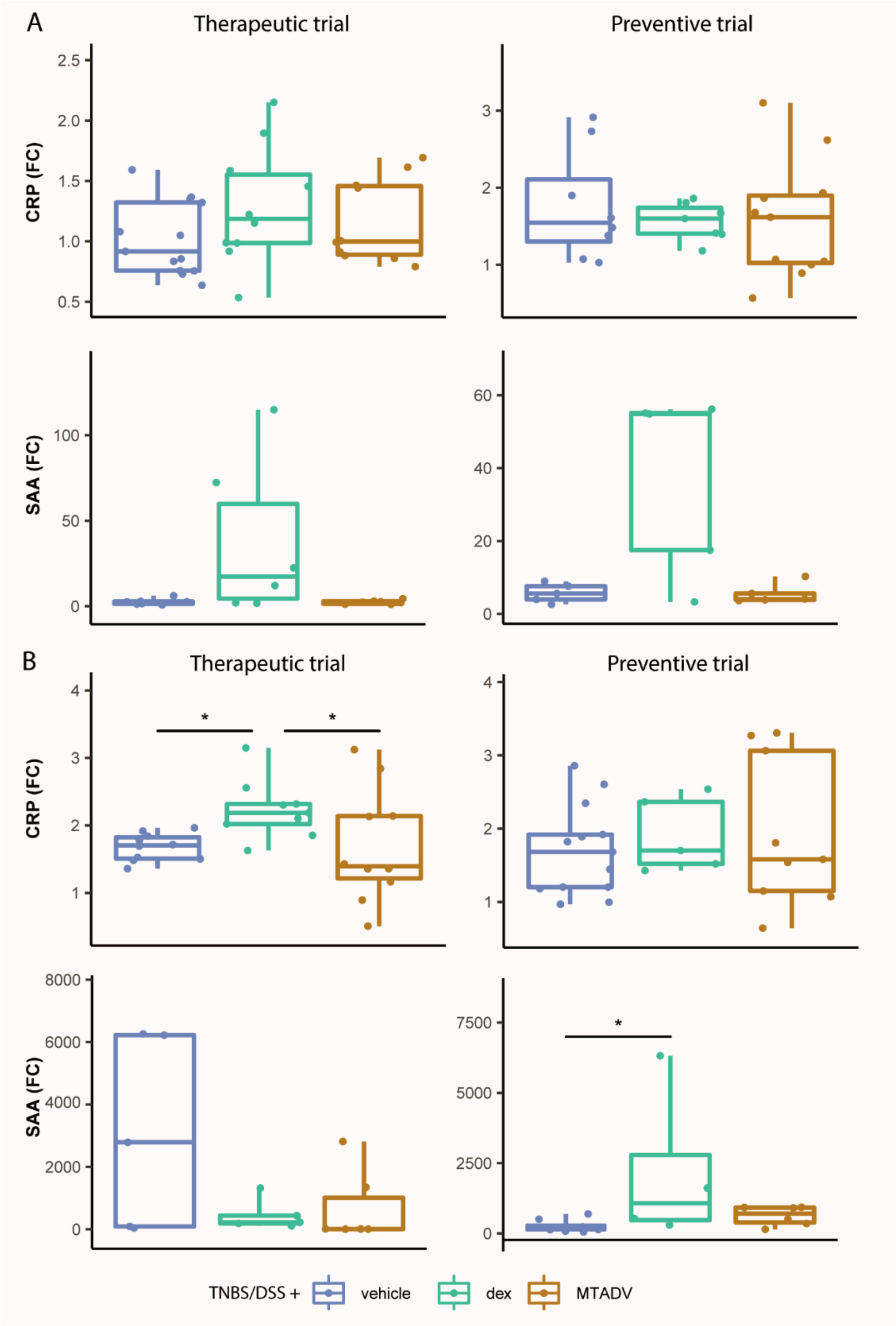

Figure S1. Concentration of plasmatic APP in each treatment group. APP protein concentration is expressed as ng/ml for CRP and ug/ml for SAA for both therapeutic and preventive trials in the TNBS (A) and DSS (B) models. Data are represented as points summarized in boxplots indicating IQR and bars indicating maximum and minimum. The median point is depicted as a bar inside the box. Treatment groups are differentiated using colours. Asterisks mark significance between the groups indicated by lines (Kruskall-Wallis test with a post-hoc Dunn's test). Significance levels are: *p-value≤0.05; **p-value≤0.01; ***p-value≤0.001. APP, acute-phase protein; CRP, C-reactive protein; dex, dexamethasone; DSS, dextran sulphate sodium; FC, fold change; IQR, interquartile range; SAA, Serum Amyloid Antigen; MTADV, 5-MER peptide methionine-threonine-alanine-aspartic acid-valine; TNBS, trinitrobenzenesulfonic acid.

Supplementary tables

Table S1. BLI raw data correlation to mRNA from inflammatory and oxidative stress markers in all the animal groups stratified by sex.

		Il10	*Tgfb1*	*Il6*	*Vegfa*	*Tjp1*	*Nos2*	*Tnfa*	*Hif1a*	*Il1b1*
**Both sexes**	pvalue	0.70	**0.04**	0.06	**0.01**	**0.02**	**0.01**	**0.032**	0.87	0.09
	rho	-0.04	**-0.19**	0.18	**-0.24**	**-0.22**	**0.25**	**0.20**	0.02	0.16
**F**	pvalue	**0.03**	0.26	**0.01**	0.96	0.36	0.25	0.072	0.32	**0.03**
	rho	**0.29**	0.15	**0.33**	0.00	-0.12	0.15	0.24	0.13	**0.29**
**M**	pvalue	0.63	**0.00**	0.81	**0.00**	**0.02**	**0.01**	0.22	0.57	0.63
	rho	0.07	**-0.43**	0.03	**-0.46**	**-0.3**	**0.34**	0.17	-0.08	0.07

Spearman correlation analyses. Red and blue colors indicate significant (p≤0.05) negative and positive correlations, respectively. BLI; Bioluminescence intensity; DSS, dextran sulfate sodium; F, female; M, male; TNBS, trinitrobenzenesulfonic acid.

Table S2. BLI raw data correlation to mRNA anti-inflammatory/pro-inflammatory markers of all the animal together and stratified by sex

		Il10/Il6	*Tgfb1/Il6*	*Il10/Tnfa*	*Tgfb1/ Tnfa*	*Il10/Il1b1*	*Tgfb1/ Il1b1*
**Both sexes**	pvalue	**0.00**	**0.02**	**0.01**	**0.00**	**0.00**	**0.02**
	rho	**-0.31**	**-0.22**	**-0.28**	**-0.25**	**-0.32**	**-0.22**
**F**	pvalue	**0.00**	**0.02**	**0.03**	**0.03**	**0.00**	**0.03**
	rho	**-0.40**	**-0.30**	**-0.32**	**-0.29**	**-0.44**	**-0.29**
**M**	pvalue	0.15	0.36	0.09	**0.00**	0.10	0.25
	rho	-0.22	-0.13	-0.26	**-0.37**	-0.25	-0.16

Spearman correlation analyses. Red and blue colors indicate significant (p≤0.05) negative and positive correlations. BLI, Bioluminescence intensity; DSS, dextran sulfate sodium; F, female; M, male; TNBS, trinitrobenzenesulfonic acid.

Table S3. BLI correlation to mRNA anti-inflammatory/pro-inflammatory markers of all the animals stratified by model and sex

				Il10/Il6	*Tgfb1/Il6*	*Il10/Tnfa*	*Tgfb1/ Tnfa*	*Il10/Il1b1*	*Tgfb1/ Il1b1*
**Both sexes**	**TNBS**	**Therapeutic**	pvalue	0.62	0.68	0.53	0.41	0.36	0.45
			rho	-0.12	-0.08	-0.15	-0.15	-0.23	-0.14
		**Preventive**	pvalue	0.62	0.68	0.53	0.41	0.36	0.45
			rho	-0.12	-0.08	-0.15	-0.15	-0.23	-0.14
	**DSS**	**Therapeutic**	pvalue	**0.00**	**0.01**	0.06	**0.04**	**0.01**	**0.03**
			rho	**-0.48**	**-0.48**	-0.36	**-0.40**	**-0.47**	**-0.41**
		**Preventive**	pvalue	0.45	**0.04**	0.39	0.46	0.83	0.81
			rho	0.17	**0.39**	-0.19	-0.15	-0.05	0.05
**F**	**TNBS**	**Therapeutic**	pvalue	0.08	0.16	0.61	0.70	**0.02**	**0.02**
			rho	-0.59	-0.37	-0.19	-0.11	**-0.77**	**-0.61**
		**Preventive**	pvalue	0.15	0.23	0.33	0.87	0.11	0.5
			rho	0.47	0.33	0.33	-0.05	0.52	0.19
	**DSS**	**Therapeutic**	pvalue	0.33	0.43	0.70	0.52	0.24	0.49
			rho	-0.27	-0.22	0.11	0.18	-0.33	-0.19
		**Preventive**	pvalue	0.14	0.09	0.43	0.24	0.69	0.44
			rho	0.48	0.5	-0.26	-0.37	-0.14	-0.24
**M**	**TNBS**	**Therapeutic**	pvalue	0.13	0.43	1.00	0.32	0.44	0.37
			rho	0.55	0.17	-0.00	-0.25	0.3	0.24
		**Preventive**	pvalue	0.91	0.89	0.86	0.52	0.61	0.90
			rho	-0.05	-0.06	-0.07	-0.22	-0.19	-0.05
	**DSS**	**Therapeutic**	pvalue	0.22	0.52	**0.03**	**0.04**	0.72	0.39
			rho	-0.37	-0.20	**-0.60**	**-0.58**	0.12	0.27
		**Preventive**	pvalue	0.44	**0.05**	0.78	0.78	0.6	0.21
			rho	0.24	**0.52**	-0.09	-0.08	0.17	0.34

Spearman correlation analyses. Red and blue colors indicate significant ($p \leq 0.05$) negative and positive correlations. BLI, Bioluminescence; DSS, dextran sulfate sodium; F, female; M, male; RD, Raw data; TNBS, trinitrobenzenesulfonic acid.

Table S4. Macroscopic evaluation measurements (median ± SD) stratified by model, trial and sex compared by treatment and between sexes in each treatment group.

				median ± SD (UMW)						p-value (Dunn's test)		
		**Gene**	**Sex**	**controls**	**p-value**	**dex**	**p-value**	**MTADV**	**p-value**	**ctl vs dex**	**ctl vs MTADV**	**dex vs MTADV**
**TNBS**	**Therapeutic**	**Spleen weight (g)**	F	0.13 ± 0.06	ns	0.04 ± 0.01	ns	0.1 ± 0.02	ns	0.007	ns	0.025
			M	0.09 ± 0.02		0.07 ± 0.02		0.09 ± 0.01		0.042	ns	0.050
		**Colon weight/ length (g/cm)**	F	0.03 ± 0.01	ns	0.03 ± 0	ns	0.04 ± 0.01	ns	ns	ns	0.050
			M	0.04 ± 0.01		0.03 ± 0.01		0.03 ± 0.01		ns	ns	ns
		**Hyperaemia**	F	0.83 ± 0.98	ns	0.4 ± 0.55	ns	0.8 ± 0.84	ns	ns	ns	ns
			M	0.71 ± 0.76		0.4 ± 0.55		0.8 ± 0.84		ns	ns	ns
		**Strictures number**	F	1.67 ± 0.82	ns	1.2 ± 0.84	ns	1.8 ± 0.84	ns	ns	ns	ns
			M	2 ± 0.82		1.4 ± 0.89		2.2 ± 1.48		ns	ns	ns
		**Wallace score**	F	3 ± 0.89	ns	1.8 ± 1.3	ns	3.6 ± 0.55	ns	ns	ns	0.015
			M	2.71 ± 0.49		1.8 ± 1.1		2.4 ± 1.34		ns	ns	ns
		**Adherences**	F	2.33 ± 0.82	ns	0.6 ± 0.55	ns	2.4 ± 0.89	0.049	0.011	ns	0.011
			M	1.57 ± 0.79		0.6 ± 0.55		1 ± 0.71		0.031	ns	ns
	**Preventive**	**Spleen weight (g)**	F	0.06 ± 0.02	ns	0.06 ± 0.02	ns	0.05 ± 0.02	ns	ns	ns	ns
			M	0.06 ± 0.02		0.06 ± 0.01		0.08 ± 0.01		ns	ns	0.050
		**Colon weight/ length (g/cm)**	F	0.03 ± 0	0.029	0.03 ± 0	ns	0.03 ± 0	0.009	0.015	ns	0.050
			M	0.04 ± 0		0.03 ± 0		0.04 ± 0		0.029	ns	0.017
		**Hyperaemia**	F	1.75 ± 1.26	ns	1.4 ± 0.55	ns	1.57 ± 0.53	ns	ns	ns	ns
			M	1.75 ± 0.96		1.25 ± 0.5		1.4 ± 0.89		ns	ns	ns
		**Strictures number**	F	2 ± 0	ns	0.6 ± 0.89	ns	1.43 ± 0.79	ns	0.013	ns	ns
			M	2.5 ± 1.29		1.5 ± 0.58		0.6 ± 0.55		ns	0.012	ns
		**Wallace score**	F	3 ± 1.5	ns	5 ± 1	ns	3 ± 1	ns	ns	ns	ns
			M	3.5 ± 1		5 ± 2		3 ± 2		ns	ns	ns
		**Adherences**	F	1.5 ± 0.71	ns	2.2 ± 1.3	ns	1.33 ± 1.03	ns	ns	ns	ns
			M	1.5 ± 0.58		2.33 ± 1.53		1.33 ± 0.58		ns	ns	ns
**DSS**	**Therapeutic**	**Spleen weight (g)**	F	0.1 ± 0.03	ns	0.04 ± 0	ns	0.08 ± 0.01	ns	0.004	ns	0.016
			M	0.08 ± 0.02		0.05 ± 0.02		0.11 ± 0.05		ns	ns	0.003
		**Colon weight/ length (g/cm)**	F	0.1 ± 0.03	0.008	0.04 ± 0	ns	0.08 ± 0.01	0.008	ns	ns	ns
			M	0.04 ± 0		0.03 ± 0.01		0.04 ± 0.01		0.031	ns	0.021
		**Hyperaemia**	F	1.4 ± 0.55	ns	1.4 ± 0.55	ns	1.6 ± 0.55	ns	ns	ns	ns
			M	1.8 ± 0.84		1 ± 0		1.6 ± 0.55		0.050	ns	ns
		**Strictures number**	F	1.6 ± 1.52	ns	2 ± 1	ns	2.4 ± 1.14	ns	ns	ns	ns
			M	1.2 ± 0.45		1 ± 1		1 ± 1		ns	ns	ns
		**Wallace score**	F	5.2 ± 2.05	ns	3.6 ± 1.34	ns	3 ± 0	ns	ns	0.032	ns
			M	4.4 ± 3.13		3 ± 0		3 ± 0		ns	ns	ns
		**Adherences**	F	1 ± 0	ns	0.4 ± 0.55	0.041	2.2 ± 0.84	ns	ns	ns	0.002
			M	2.2 ± 1.1		1.4 ± 0.55		2.4 ± 0.55		ns	ns	ns
	**Preventive**	**Spleen weight (g)**	F	0.1 ± 0.06	ns	0.11 ± 0.01	ns	0.11 ± 0.03	ns	ns	ns	ns
			M	0.13 ± 0.05		0.13 ± 0.04		0.12 ± 0.04		ns	ns	ns
		**Colon weight/ length (g/cm)**	F	0.03 ± 0.01	ns	0.03 ± 0	ns	0.03 ± 0	ns	ns	ns	ns
			M	0.03 ± 0.01		0.03 ± 0		0.03 ± 0		ns	ns	ns
		**Hyperaemia**	F	1.17 ± 0.41	ns	1.33 ± 0.58	ns	1.8 ± 0.84	ns	ns	ns	ns
			M	1.57 ± 0.98		0.67 ± 0.58		1.2 ± 0.45		ns	ns	ns
		**Strictures number**	F	0 ± 0	ns	0 ± 0	ns	0.4 ± 0.89	ns	ns	ns	ns
			M	0.43 ± 0.79		1 ± 0		0 ± 0		ns	ns	0.010
		**Wallace score**	F	8 ± 1.79	0.016	5.67 ± 3.21	ns	5.6 ± 1.52	ns	ns	0.050	ns
			M	3.71 ± 2.5		5.67 ± 2.52		5.2 ± 1.79		ns	ns	ns
		**Adherences**	F	1.67 ± 1.03	ns	2 ± 0	ns	1.6 ± 0.89	ns	ns	ns	ns
			M	1.86 ± 0.38		2.33 ± 0.58		0.8 ± 0.45		ns	0.010	0.004

Data are given as median ± sd and the result of the statistical test between groups is given as p-value. DSS, dextran sulfate sodium; F, female; M, male; MTADV, 5-MER peptide methionine-threonine-alanine-aspartic acid-valine; ns, non-significant; SD, standard deviation; TNBS, trinitrobenzenesulfonic acid.

Table S5. mRNA expression (FC) of inflammatory and oxidative stress markers stratified by both trial and sex in the TNBS models compared by treatment and between sexes in each treatment group.

				median (IQR; UMW)						p-value (Dunn's test)		
		**Gene**	**Sex**	**controls**	**p-value**	**dex**	**p-value**	**MTADV**	**p-value**	**ctl vs dex**	**ctl vs MTADV**	**dex vs MTADV**
**TNBS**	**Therapeutic**	***Hif1a***	F	1.1 (1-1.3)	ns	1.2 (1.1-1.4)	ns	1.6 (1.4-2.5)	ns	ns	ns	ns
			M	1.3 (1-1.4)		1.2 (1-1.4)		1.4 (1.3-1.4)		ns	ns	ns
		Il10	F	0.8 (0.6-0.9)	ns	0.4 (0.4-0.7)	ns	1 (0.7-1.1)	ns	ns	ns	ns
			M	0.4 (0.4-0.5)		0.6 (0.6-0.7)		0.8 (0.5-1.2)		ns	ns	ns
		Il6	F	1.4 (1-1.5)	ns	0.7 (0.6-0.8)	ns	36.5 (1.7-45)	ns	ns	ns	0.003
			M	0.7 (0.6-1)		0.8 (0.6-0.9)		1.6 (1.2-1.9)		ns	0.050	0.040
		Nos2	F	0.8 (0.6-1.3)	0.012	0.5 (0.4-0.6)	ns	1.2 (0.5-1.5)	ns	ns	ns	ns
			M	0.3 (0.3-0.4)		0.4 (0.4-0.4)		0.8 (0.6-0.9)		ns	0.027	ns
		Tgfb1	F	1.4 (1.3-2)	ns	1.3 (1.2-1.4)	ns	2.7 (2.3-2.8)	ns	ns	ns	0.009
			M	1.4 (1.1-1.5)		1.3 (1.1-1.4)		2.3 (2.2-2.4)		ns	0.008	0.006
		Tnfa	F	0.9 (0.8-1.2)	0.017	0.7 (0.6-0.8)	ns	1 (0.9-1)	ns	ns	ns	0.046
			M	0.6 (0.5-0.6)		0.5 (0.4-0.8)		0.8 (0.7-1.1)		ns	0.040	ns
		Vegfa	F	1.4 (1.2-1.6)	ns	1.4 (1.3-1.6)	ns	1.5 (1.5-1.6)	ns	ns	ns	ns
			M	1.3 (1.1-1.4)		1.5 (1.1-1.8)		1.5 (1.4-1.5)		ns	ns	ns
		Tpj1	F	1.3 (1.1-1.5)	ns	1.5 (1.5-1.8)	ns	1.4 (1.3-1.7)	ns	ns	ns	ns
			M	1.3 (1.3-1.7)		1.7 (1-1.7)		1.5 (1.5-1.7)		ns	ns	ns
		Il1b1	F	1.4 (0.8-1.6)	ns	1 (0.6-1.2)	ns	4.2 (1.4-24.3)	ns	ns	ns	0.019
			M	0.4 (0.4-0.6)		0.4 (0.3-1.1)		2.1 (0.9-2.2)		ns	0.038	0.028
	**Preventive**	***Hif1a***	F	1.1 (1.1-1.3)	ns	1.7 (1.4-1.8)	ns	2.7 (2.1-2.9)	ns	ns	0.001	0.018
			M	1.6 (1.5-1.8)		2.1 (1.5-2.2)		2.3 (2.1-3.3)		ns	ns	ns
		Il10	F	0.8 (0.8-0.8)	ns	0.4 (0.3-0.5)	ns	0.8 (0.7-0.9)	ns	ns	ns	0.018
			M	0.6 (0.5-0.8)		0.8 (0.6-1)		1 (0.9-1.2)		ns	ns	ns
		Il6	F	1.7 (1.6-1.9)	ns	9.6 (4.7-21.7)	ns	21.8 (5.8-81.6)	ns	ns	0.050	ns
			M	9.6 (0.8-46.4)		84.6 (54.3-93.8)		52.9 (1.9-128)		ns	ns	ns
		Nos2	F	0.7 (0.5-0.9)	0.002	1.4 (1.2-1.6)	ns	0.8 (0.8-2)	0.039	0.030	ns	ns
			M	2 (1.5-2.5)		2.3 (1.3-2.5)		3.3 (2.1-3.5)		ns	ns	ns
		Tgfb1	F	1.5 (1.4-1.5)	ns	1.9 (1.8-1.9)	ns	2.6 (1.7-2.8)	ns	ns	0.041	ns
			M	1.3 (1.1-1.6)		1.8 (1.6-2.2)		1.7 (1.6-3.9)		ns	ns	ns
		Tnfa	F	0.8 (0.7-0.9)	ns	1.3 (1.3-1.4)	ns	1.3 (1.2-1.9)	ns	ns	ns	ns
			M	1.1 (1-1.4)		1.8 (1.2-2.4)		1.6 (1.6-2)		ns	ns	ns
		Vegfa	F	1.2 (1.1-1.2)	ns	1.7 (1.2-1.7)	ns	1.9 (1.5-2.1)	ns	ns	0.050	ns
			M	1.4 (1.1-1.7)		1.7 (1.5-1.8)		1.5 (1.2-2.2)		ns	ns	ns
		Tpj1	F	1.4 (1.3-1.5)	ns	1.1 (1.1-1.1)	ns	1.6 (1.3-1.8)	ns	ns	ns	0.024
			M	1.3 (1-1.5)		1.4 (1.3-1.6)		1.6 (1.5-1.7)		ns	ns	ns
		Il1b1	F	1 (0.7-1.6)	ns	4.9 (3.3-28.2)	ns	4.9 (2.8-12.6)	ns	0.016	0.024	ns
			M	7 (1.3-32)		7.5 (6.8-33.9)		2.8 (2.6-30.7)		ns	ns	ns

Data are given as median (IQR) and the result of the statistical test between groups is given as p-value. F, female; FC, Fold-change; IQR, interquartile range; M, male; MTADV, 5-MER peptide methionine-threonine-alanine-aspartic acid-valine; ns, non-significant, TNBS, trinitrobenzenesulfonic acid, UMW; U Mann Whitney.

Table S6. mRNA expression (FC) of inflammatory and oxidative stress markers stratified by both trial and sex in the DSS models compared by treatment and between sexes in each treatment group.

				median (IQR; UMW)						p-value (Dunn's test)		
		**Gene**	**Sex**	**controls**	**p-value**	**dex**	**p-value**	**MTADV**	**p-value**	**ctl vs dex**	**ctl vs MTADV**	**dex vs MTADV**
**DSS**	**Therapeutic**	***Hif1a***	F	2.6 (2.1-3)	ns	1.8 (1.3-2.3)	ns	1.1 (0.8-1.2)	ns	ns	0.006	ns
			M	1.6 (1.6-2.9)		2.2 (1.7-2.3)		1.9 (1.7-2.3)		ns	ns	ns
		Il10	F	1.3 (1.2-1.3)	ns	1.5 (1.2-1.9)	ns	1.3 (1.1-1.4)	ns	ns	ns	ns
			M	1.5 (1-3.5)		1.3 (1.2-1.4)		0.8 (0.8-0.8)		ns	0.040	ns
		Il6	F	2.8 (2.6-4.8)	0.009	9.4 (4.6-13.1)	ns	0.9 (0.8-1.8)	0.001	ns	ns	0.009
			M	69.9 (30.3-83)		29.5 (25.8-41.4)		30.4 (25.1-33.7)		ns	ns	ns
		Nos2	F	1.4 (0.9-2)	0.009	0.5 (0.5-0.9)	ns	2.6 (2.1-2.6)	ns	ns	ns	0.004
			M	4.5 (3.4-6.5)		1.7 (1.4-1.7)		8.2 (5.3-12.2)		0.048	ns	0.007
		Tgfb1	F	1.6 (1.5-1.6)	ns	1.4 (1.4-1.5)	ns	1.2 (1.2-1.6)	ns	ns	ns	ns
			M	1.3 (0.8-1.5)		1.3 (1.2-1.9)		1.3 (0.7-1.3)		ns	ns	ns
		Tnfa	F	1.7 (1.6-2.7)	ns	0.9 (0.7-1.1)	ns	1.5 (1.4-1.5)	0.013	0.002	ns	0.024
			M	2.2 (2-2.7)		1.5 (0.9-1.6)		3.1 (2.9-3.6)		ns	ns	0.004
		Vegfa	F	1.1 (1.1-1.2)	0.017	2 (1.7-2)	ns	0.8 (0.8-1.1)	ns	0.034	ns	0.003
			M	0.8 (0.7-0.9)		1.1 (1-1.2)		0.9 (0.8-0.9)		0.011	ns	0.034
		Tpj1	F	1.6 (1.5-1.7)	0.017	1.7 (1.6-1.7)	ns	0.8 (0.7-1)	ns	ns	0.016	0.004
			M	0.9 (0.9-1)		1.4 (1.3-1.5)		0.7 (0.7-0.7)		ns	ns	0.001
		Il1b1	F	3.3 (2.4-4.5)	0.029	3.2 (2.7-4.4)	0.031	1.8 (1.5-2.5)	0.004	ns	ns	ns
			M	26.1 (16.7-37.4)		21.5 (10.1-39)		14.1 (8.5-14.8)		ns	ns	ns
	**Preventive**	***Hif1a***	F	1.1 (1.1-1.2)	ns	1.4 (1.2-1.7)	0.024	1.3 (1.2-1.6)	0.049	ns	ns	ns
			M	1.6 (1.4-1.7)		2.9 (2.9-3.5)		2.4 (1.9-2.5)		0.003	0.029	ns
		Il10	F	0.8 (0.7-0.9)	ns	1.4 (1.3-1.5)	ns	0.8 (0.8-1)	ns	ns	ns	ns
			M	1.1 (0.5-1.8)		NA (NA-NA)		0.8 (0.7-1.1)		NA	ns	NA
		Il6	F	16.2 (12.4-18.1)	ns	33.9 (33-45.2)	0.035	21.3 (16.6-40.3)	0.034	ns	ns	ns
			M	59 (21.8-79.5)		701 (445.9-2012.7)		148.5 (116-167.9)		0.002	ns	ns
		Nos2	F	2.6 (2.2-2.7)	0.009	3.7 (2.7-3.8)	ns	3.2 (2.9-5)	ns	ns	ns	ns
			M	3.3 (2.5-4.7)		1.4 (1.4-1.8)		4.4 (4-5.2)		ns	ns	0.030
		Tgfb1	F	1.3 (1.2-1.3)	ns	1.4 (1.2-1.5)	0.013	1.7 (1.6-1.8)	ns	ns	0.033	ns
			M	1.4 (1.2-1.8)		2.5 (2.2-2.6)		2.2 (1.9-2.4)		0.013	0.044	ns
		Tnfa	F	2.4 (1.8-2.5)	ns	2.6 (2.4-2.7)	ns	2.1 (2-2.3)	0.015	ns	ns	ns
			M	2.5 (1.9-3.1)		4.1 (3.2-4.1)		3.5 (3.4-5.2)		ns	0.031	ns
		Vegfa	F	0.9 (0.8-0.9)	ns	0.7 (0.7-0.8)	0.003	0.9 (0.9-1)	ns	ns	ns	0.02
			M	0.7 (0.7-0.8)		1.8 (1.4-1.9)		0.9 (0.8-0.9)		0.003	ns	ns
		Tpj1	F	0.9 (0.9-1)	ns	0.5 (0.5-0.5)	ns	0.6 (0.6-0.6)	ns	0.002	ns	ns
			M	0.6 (0.6-0.9)		0.6 (0.6-0.7)		0.8 (0.7-0.8)		ns	ns	ns
		Il1b1	F	6 (4.7-7.6)	ns	7.7 (7.4-11.8)	ns	12.8 (10.9-15.7)	ns	ns	ns	ns
			M	16.3 (9.4-29.7)		34.3 (26.5-38.7)		27 (26.7-34.3)		ns	ns	ns

Data are given as median (IQR) and the result of the statistical test between groups is given as p-value. DSS, dextran sulfate sodium; F, female; IQR, interquartile range; M, male; MTADV, 5-MER peptide methionine-threonine-alanine-aspartic acid-valine; ns, non-significant, UMW; U Mann Whitney.

Table S7. FCs of CRP and SAA stratified by sex in preventive and therapeutic approaches compared between treatments and between sex in each treatment group.

				median (IQR; UMW)						p-value (Dunn's test)		
		APP	Sex	controls	p-value	dex	p-value	MTADV	p-value	ctl vs dex	ctl vs MTADV	dex vs MTADV
TNBS	Therapeutic	CRP	F	1.0 (0.8-1.3)	ns	1.5 (1.2-1.6)	ns	1.0 (0.9-1.5)	ns	ns	ns	ns
			M	0.9 (0.8-1.2)		1.0 (0.9-1.2)		1.0 (0.9-1.4)		ns	ns	ns
		SAA	F	1.6 (1.2-2.2)	ns	22.4 (17.3-47.4)	ns	2.1 (1.6-2.4)	ns	0.037	ns	ns
			M	2.6 (2.6-6.2)		2.0 (1.9-58.4)		3.0 (2.1-3.7)		ns	ns	ns
	Preventive	CRP	F	1.2 (1.1-1.4)	ns	1.6 (1.4-1.7)	ns	1.0 (0.9-1.7)	ns	ns	ns	ns
			M	2.3 (1.8-2.8)		1.8 (1.6-1.8)		1.7 (1.6-2.6)		ns	ns	ns
		SAA	F	4.0 (3.3-4.8)	ns	17.5 (10.4-36.3)	ns	7.2 (4-18.1)	ns	ns	ns	ns
			M	8.9 (8.3-2988.3)		56.2 (55.5-472.1)		4.7 (4.3-5.2)		ns	ns	0.044
DSS	Therapeutic	CRP	F	1.7 (1.5-1.8)	ns	2.1 (1.9-2.3)	ns	1.2 (0.9-1.4)	0.001	ns	0.048	0.001
			M	1.7 (1.5-1.9)		2.6 (2.2-3.1)		2.1 (2.1-2.8)		0.009	ns	ns
		SAA	F	68.7 (56.8-80.6)	0.033	189.5 (151.0-315.8)	ns	9.6 (7.6-11.9)	0.016	ns	ns	0.009
			M	6224.3 (4508.1-6242.9)		1322.8 (775.5-3163.9)		2080.7 (1713.5-2447.8)		ns	ns	ns
	Preventive	CRP	F	1.2 (1-1.2)	ns	1.5 (1.5-1.6)	ns	1.4 (1.1-2.0)	ns	ns	ns	ns
			M	1.9 (1.8-2.3)		2.5 (2.4-2.5)		1.8 (1.5-3.1)		ns	ns	ns
		SAA	F	134.5 (79.1-140.6)	ns	955.0 (626.5-1283.5)	ns	924.3 (922.0-926.5)	ns	ns	ns	ns
			M	367.3 (224.2-853.4)		3424.5 (1977.1-4871.9)		427.7 (300.7-603.5)		ns	ns	ns

Data are given as median (interquartile range) and the result of the statistical test between groups is given as p-value. DSS, dextran sulfate sodium; F, female; FC, fold change; IQR, interquartile range; M, male; MTADV, 5-MER peptide methionine-threonine-alanine-aspartic acid-valine; ns, non-significant, TNBS, trinitrobenzenesulfonic acid.

Table S8. Correlation between inflammatory and oxidative stress mRNA expression in both therapeutic and preventive approaches in the TNBS model in females.

	Hif1a	*Il10*	*Il6*	*Nos2*	*Tgfb1*	*Tnfa*	*Vegfa*	*Tjp1*	*Il1b1*	
Hif1a		0.21	0.42	-0.19	**0.64**	-0.00	**0.77**	**0.51**	0.17	**Therapeutic**
Il10	0.51		-0.21	0.60	0.26	**0.7**	-0.30	-0.04	**0.75**	
Il6	**0.64**	-0.11		0.26	0.42	0.39	0.43	-0.04	**0.59**	
Nos2	0.34	0.29	0.33		-0.16	**0.61**	-0.27	-0.41	**0.55**	
Tgfb1	**0.62**	0.25	**0.73**	0.38		0.11	0.31	0.17	0.23	
Tnfa	0.50	0.04	**0.73**	0.41	0.49		-0.28	-0.47	**0.78**	
Vegfa	**0.68**	0.01	**0.83**	0.24	**0.56**	**0.62**		0.36	-0.16	
Tjp1	0.46	0.57	0.10	-0.31	0.36	-0.18	0.29		-0.20	
Il1b1	**0.64**	-0.19	**0.86**	**0.66**	**0.74**	**0.66**	**0.71**	-0.11		
	**Preventive**									

Spearman linear regression analyses. Red and blue colors indicate significant (p≤0.05) negative and positive correlations respectively. The grey diagonal division separates therapeutic (top), from preventive (bottom) correlations. TNBS, trinitrobenzenesulfonic acid.

Table S9. Correlation between inflammatory and oxidative stress mRNA expression in both therapeutic and preventive approaches in the TNBS model in males.

	Hif1a	*Il10*	*Il6*	*Nos2*	*Tgfb1*	*Tnfa*	*Vegfa*	*Tjp1*	*Il1b1*	
Hif1a		0.14	0.36	0.23	0.44	0.09	0.42	**0.58**	0.01	**Therapeutic**
Il10	0.49		0.23	0.48	0.32	0.56	0.01	-0.27	**0.85**	
Il6	**0.81**	0.52		0.26	**0.92**	**0.64**	**0.65**	**0.81**	0.38	
Nos2	0.23	0.39	0.12		0.33	0.48	0.33	-0.02	0.24	
Tgfb1	**0.88**	0.37	**0.87**	0.41		**0.52**	0.34	**0.62**	0.40	
Tnfa	**0.63**	0.28	**0.71**	**0.67**	**0.75**		-0.27	-0.01	**0.77**	
Vegfa	**0.79**	0.36	**0.93**	0.27	**0.89**	**0.67**		**0.63**	-0.49	
Tjp1	0.19	0.28	0.16	-0.04	0.18	0.02	0.24		-0.19	
Il1b1	0.57	0.15	**0.86**	0.32	**0.73**	**0.62**	**0.87**	-0.08		
	**Preventive**									

Spearman linear regression analyses. Red and blue colors indicate significant (p≤0.05) negative and positive correlations respectively. The grey diagonal division separates therapeutic (top), from preventive (bottom) correlations. TNBS, trinitrobenzenesulfonic acid.

Table S10. Correlation between inflammatory and oxidative stress mRNA expression in both therapeutic and preventive approaches in the DSS model in females.

	Hif1a	***Il10***	***Il6***	***Nos2***	***Tgfb1***	***Tnfa***	***Vegfa***	***Tjp1***	***Il1b1***	
Hif1a		0.22	**0.61**	-0.50	**0.63**	0.07	0.45	**0.67**	**0.64**	**Therapeutic**
Il10	-0.07		**0.60**	-0.41	0.46	-0.38	**0.59**	0.16	0.48	
Il6	0.29	0.26		**-0.62**	0.36	-0.38	**0.67**	**0.61**	**0.89**	
Nos2	0.01	0.03	0.39		-0.19	**0.65**	**-0.60**	**-0.75**	-0.46	
Tgfb1	0.45	-0.23	0.11	-0.25		0.38	0.32	0.35	0.39	
Tnfa	0.06	-0.01	0.19	**0.71**	-0.06		-0.45	-0.22	-0.23	
Vegfa	-0.03	-0.35	**-0.75**	-0.18	0.50	-0.18		**0.67**	0.43	
Tjp1	-0.08	-0.50	**-0.60**	-0.45	-0.25	-0.14	0.43		0.40	
Il1b1	0.30	-0.09	0.50	**0.68**	0.20	0.47	-0.15	-0.27		
	**Preventive**									

Spearman linear regression analyses. Red and blue colors indicate significant ($p \leq 0.05$) negative and positive correlations respectively. The grey diagonal division separates therapeutic (top), from preventive (bottom) correlations. DSS, dextran sulfate sodium.

Table S11. Correlation between inflammatory and oxidative stress mRNA expression in both therapeutic and preventive approaches in the DSS model in males.

	Hif1a	***Il10***	***Il6***	***Nos2***	***Tgfb1***	***Tnfa***	***Vegfa***	***Tjp1***	***Il1b1***	
Hif1a		0.29	0.09	-0.23	0.05	-0.17	0.15	0.35	0.33	**Therapeutic**
Il10	-0.19		0.25	-0.10	0.46	-0.13	0.36	0.41	**0.56**	
Il6	**0.70**	0.47		0.22	0.23	0.17	-0.29	-0.23	**0.67**	
Nos2	-0.26	-0.48	-0.46		-0.13	**0.96**	-0.41	**-0.69**	0.18	
Tgfb1	**0.68**	0.43	**0.84**	-0.38		-0.06	**0.55**	0.37	**0.64**	
Tnfa	**0.53**	-0.13	0.40	0.32	0.45		-0.29	**-0.67**	0.18	
Vegfa	**0.65**	0.06	**0.78**	-0.29	**0.78**	**0.66**		**0.7**	0.17	
Tjp1	0.23	**-0.71**	-0.25	0.47	-0.04	0.18	0.00		0.15	
Il1b1	0.45	**0.59**	**0.57**	-0.36	**0.66**	0.49	0.46	-0.33		
	**Preventive**									

Spearman linear regression analyses. Red and blue colors indicate significant ($p \leq 0.05$) negative and positive correlations respectively. The grey diagonal division separates therapeutic (top), from preventive (bottom) correlations. DSS, dextran sulfate sodium.

Table S12. Correlation between plasmatic protein levels of CRP and SAA, and mRNA expression of inflammation markers separated by animal model and design.

				Il10	*Tgfb1*	*Il6*	*Vegfa*	*Tjp1*	*Nos2*	*Tnfa*	*Hif1a*	*Il1b1*
**CRP**	**TNBS**	**Therapeutic**	p-value	0.34	0.71	0.10	0.57	0.64	0.40	0.68	0.10	0.37
			rho	0.22	0.07	-0.31	-0.10	-0.08	0.15	0.07	0.29	0.17
		**Preventive**	p-value	0.15	0.81	0.29	0.24	0.81	0.07	0.96	0.35	0.12
			rho	0.32	-0.05	0.22	0.23	-0.05	0.36	0.01	0.18	0.31
	**DSS**	**Therapeutic**	p-value	0.50	0.91	**6E-3**	0.20	0.17	0.93	0.77	**0.02**	**2E-3**
			rho	0.13	-0.02	**0.49**	0.24	0.26	-0.02	0.05	**0.43**	**0.55**
		**Preventive**	p-value	0.81	0.14	**0.04**	0.21	0.37	0.64	0.69	**3E-3**	**0.04**
			rho	-0.05	0.30	**0.41**	-0.26	-0.18	0.10	0.08	**0.56**	**0.41**
**SAA**	**TNBS**	**Therapeutic**	p-value	0.60	0.62	0.10	**0.04**	0.32	0.13	0.17	0.42	0.74
			rho	0.16	-0.12	-0.43	**-0.47**	-0.23	-0.35	0.32	-0.19	0.09
		**Preventive**	p-value	0.80	0.43	**3E-3**	**0.04**	0.67	0.31	0.32	0.40	**8E-4**
			rho	-0.07	0.20	**0.67**	**0.48**	0.11	0.25	0.25	0.21	**0.73**
	**DSS**	**Therapeutic**	p-value	0.72	0.77	**0.00**	0.69	0.74	0.06	0.06	**0.04**	**0.00**
			rho	-0.10	-0.08	**0.87**	-0.10	0.09	0.47	0.46	**0.50**	**0.91**
		**Preventive**	p-value	0.70	0.32	0.06	0.49	0.09	0.60	0.46	0.31	0.19
			rho	0.11	0.25	0.45	0.17	-0.41	0.13	0.19	0.25	0.32

Spearman linear regression analyses. Red and blue colors indicate significant ($p \leq 0.05$) negative and positive correlations respectively. CRP, C-reactive protein; DSS, dextran sulfate sodium; SAA, Serum amyloid antigen; TNBS, trinitrobenzenesulfonic acid.

Table S13. Correlation between plasmatic CRP and SAA, and mRNA expression of inflammation markers separated by model, trial and sex.

					Il10	*Tgfb1*	*Il6*	*Vegfa*	*Tjp1*	*Nos2*	*Tnfa*	*Hif1a*	*Il1b1*
CRP	F	TNBS	Therapeutic	p-value	0.71	0.7	0.27	1.00	0.96	0.56	0.46	0.53	0.64
				rho	0.14	0.11	-0.29	0.00	0.01	-0.16	0.20	0.17	0.14
			Preventive	p-value	0.52	0.37	0.80	0.91	0.31	0.17	0.43	0.71	0.40
				rho	-0.22	-0.25	-0.07	0.03	-0.28	0.38	-0.22	-0.10	0.24
		DSS	Therapeutic	p-value	0.35	0.37	**0.01**	**1E-3**	**0.00**	**8E-5**	0.06	**8E-3**	0.07
				rho	0.26	0.25	**0.63**	**0.78**	**0.87**	**-0.84**	-0.50	**0.66**	0.48
			Preventive	p-value	0.47	0.18	**0.03**	0.53	0.26	0.14	0.08	0.1	0.11
				rho	0.24	0.41	**0.64**	-0.20	-0.36	0.45	0.52	0.5	0.49
	M	TNBS	Therapeutic	p-value	0.79	0.98	0.28	0.72	0.63	0.37	0.59	0.08	0.65
				rho	0.1	0.01	-0.34	-0.09	-0.13	0.23	-0.14	0.43	0.12
			Preventive	p-value	0.12	0.77	0.31	0.25	0.54	0.64	0.99	0.20	0.32
				rho	0.50	0.10	0.34	0.36	0.20	-0.15	-0.01	0.40	0.31
		DSS	Therapeutic	p-value	0.52	0.43	0.66	**0.02**	0.16	0.79	0.85	0.49	0.55
				rho	0.18	0.22	-0.13	**0.61**	0.38	-0.08	0.05	0.19	0.17
			Preventive	p-value	0.59	0.41	0.89	0.18	0.39	0.55	0.21	0.67	0.77
				rho	-0.17	-0.24	0.04	-0.38	-0.25	-0.17	-0.36	0.13	-0.09
SAA	F	TNBS	Therapeutic	p-value	0.50	0.27	**0.02**	0.25	0.49	0.25	0.88	0.49	0.84
				rho	0.32	-0.42	**-0.77**	-0.43	-0.27	-0.43	0.07	-0.27	0.10
			Preventive	p-value	0.70	0.08	**0.02**	**0.04**	0.71	**0.02**	**0.01**	0.22	**0.01**
				rho	-0.17	0.59	**0.76**	**0.66**	-0.14	**0.76**	**0.78**	0.43	**0.77**
		DSS	Therapeutic	p-value	0.18	0.31	**3E-3**	**0.03**	**0.01**	**0.02**	0.15	0.12	**3E-3**
				rho	0.50	0.38	**0.88**	**0.73**	**0.82**	**-0.78**	-0.53	0.56	**0.88**
			Preventive	p-value	0.50	0.50	**0.04**	0.75	0.10	0.06	0.36	0.98	**0.02**
				rho	0.32	0.29	**0.76**	-0.14	-0.64	0.71	0.38	-0.02	**0.83**
	M	TNBS	Therapeutic	p-value	0.80	0.63	0.66	**0.04**	0.69	0.20	0.17	0.71	0.54
				rho	0.14	0.16	-0.21	**-0.64**	-0.14	-0.42	0.45	-0.13	0.22
			Preventive	p-value	0.84	1.00	0.15	0.24	0.30	**0.05**	0.39	0.98	0.11
				rho	-0.11	0.00	0.57	0.48	0.43	**-0.74**	-0.36	-0.02	0.62
		DSS	Therapeutic	p-value	0.75	0.30	0.15	0.33	0.88	0.46	0.54	0.62	0.14
				rho	0.14	0.43	0.57	-0.40	-0.07	0.31	0.26	0.21	0.64
			Preventive	p-value	0.70	0.84	0.22	0.41	0.84	0.43	1.00	0.22	0.79
				rho	-0.17	0.08	0.43	0.30	-0.08	-0.28	0.01	0.43	-0.10

Spearman linear regression analyses. Red and blue colors indicate significant ($p \leq 0.05$) negative and positive correlations respectively. CRP, C-reactive protein; DSS, dextran sulfate sodium; SAA, Serum amyloid antigen; TNBS, trinitrobenzenesulfonic acid.

Table S14: Macroscopic colon examination score (Wallace).

Score	Macroscopic colon examination (Wallace)
0	no damage
1	hyperaemia without ulcers
2	hyperaemia and wall thickening without ulcers
3	one ulceration site without wall thickening
4	two or more ulceration sites
5	0.5 cm extension of inflammation or major damage
6	1 cm extension of inflammation or severe damage
**the score was then increased by 1 up to a maximal score of 10**	
10*	*every 0.5 cm of damage
	*stricture presence
	*presence of mild or severe adhesion respectively
